# The Mediating Role of Self-Regulation and Artificial Intelligence Awareness in the Effect of Individual Entrepreneurship Tendencies on Learning Agility in High School Students

**DOI:** 10.3390/bs16060973

**Published:** 2026-06-11

**Authors:** Merve Coşgun Demirdağ, Najwa Salem Albeladi, Juan Gómez-Salgado, Murat Yıldırım

**Affiliations:** 1 Ministry of National Education, Ağrı 04100, Türkiye; 2 Communications Skills Department, Faculty of Science and Arts—Rabigh, King Abdulaziz University, Jeddah 21589, Saudi Arabia; 3 Department of Sociology, Social Work and Public Health, Faculty of Labour Sciences, University of Huelva, 21071 Huelva, Spain; 4 Safety and Health Postgraduate Program, Universidad Espíritu Santo, Guayaquil 092301, Ecuador; 5 Department of Psychology, Faculty of Science and Letters, Ağrı Ibrahim Cecen University, Ağrı 04100, Türkiye; 6 Psychology Research Center, Khazar University, Baku AZ1096, Azerbaijan

**Keywords:** individual entrepreneurial tendencies, self-regulation, artificial intelligence awareness, learning agility, high school students, serial mediation

## Abstract

Learning agility is considered a key competence for adapting to rapidly changing educational and technological environments. Although entrepreneurial tendencies have been associated with adaptive learning outcomes, the psychological mechanisms underlying this relationship remain insufficiently understood. This study examined whether self-regulation and artificial intelligence (AI) awareness sequentially mediate the relationship between individual entrepreneurial tendencies and learning agility among high school students. The study involved 564 high school students (55% girls, 45% boys; aged 14–19 years, M = 17.02, SD = 1.28) from two public schools in Türkiye. Participants completed validated measures of entrepreneurial tendencies, self-regulation, AI awareness, and learning agility. The hypothesized serial mediation model was tested using PROCESS Macro Model 6. Entrepreneurial tendencies were positively associated with learning agility both directly and indirectly. Self-regulation emerged as a significant independent mediator, and a significant sequential mediation pathway was identified through self-regulation and AI awareness. The findings suggest that entrepreneurial tendencies are associated with higher levels of self-regulation and AI awareness, which are in turn associated with learning agility. The results highlight the importance of self-regulation and AI awareness as factors associated with the relationship between entrepreneurial tendencies and learning agility. Educational practices that foster entrepreneurship, self-regulation, and AI awareness may support students’ adaptability and readiness for rapidly evolving digital learning environments.

## 1. Introduction

The rapid changes happening on a global scale make everyone and every organization rethink their former understanding of success. The view is particularly acute in education, and raising generations of students able to cope with the uncertainties of tomorrow is an increasingly urgent priority (Li & Chalermvongsavej, 2025). In today’s rapidly changing environment, it is no longer sufficient for individuals to merely acquire existing knowledge. The critical competency that has emerged is learning agility: the ability to quickly extract new knowledge and skills from experience, apply them effectively to novel situations, and enhance outcomes through ongoing reflective learning (Milani et al., 2024).

Learning agility is defined as the desire and ability to acquire new skills in order to work successfully with demanding and unfamiliar conditions; it includes four key dimensions: mental agility, human agility, change agility, and outcome agility (Alshammari & Alshammari, 2026). This concept, which has a firm correlation with academic success, professional continuity, and reduced burnout, is a fundamental aspect of lifelong learning skills—now particularly when labor markets are being reshaped by digitalization and automation (Li & Chalermvongsavej, 2025).

In this context of continual change, individuals are encouraged to adopt an entrepreneurial mindset, which is considered to be actively seeking solutions and identifying opportunities rather than merely reacting to environmental shifts. Entrepreneurship extends beyond the mere act of undertaking business activities; it represents a process of commercialization and a behavioral orientation defined by risk-taking, innovation, and proactive engagement (Liu et al., 2002). The personal entrepreneurial orientation shows the extent to which students can influence their own development and take initiative about solutions when facing uncertainty, and it is considered one of the major driving forces for learning agility (Hakala, 2011). Entrepreneurial learning is inherently experimental: Entrepreneurs adopt an attitude towards making mistakes as a potential learning resource and thereby develop their “learning to learn” competence (Cope, 2005). It has been found that those who have high levels of entrepreneurial tendencies are often more flexible in the matching process, updating mental models and market needs (Hakala, 2011). However, how entrepreneurial predispositions are translated into learning agility is not necessarily a linear process but depends on the conscious effort of individuals in managing their personal learning processes and the ways they make use of current technologies (Fischer et al., 2022).

Self-regulation is the control of thoughts, emotions and actions in a planned cyclical manner to achieve long-term goals (Seufert, 2018). Self-regulation, which is an essential skill to have for becoming a strategic learner, is composed of the pre-thinking (planning), performance, and self-reflection stages (Sáez-Delgado et al., 2022). Students with strong self-regulated skills can manage the learning process differently by setting their own goals, controlling and monitoring their progress, and being adaptable in their environments by adjusting strategies in case of failure (Zheng et al., 2023). The importance of self-regulation is especially salient in computer-based and digital learning environments, given the high level of student control and availability for self-determined learning in these (Winters et al., 2008). With the ability to exercise self-regulation skills, students who have an interest in business create for themselves the opportunities to deploy their cognitive resources when confronted with complex challenges and escalate the learning pace (Mardiana, 2025). In this way, self-regulation plays a dual role in the process of connecting entrepreneurial propensity with learning agility (Weinstein et al., 2011): it works both as “motor” and “glue”.

Aside from cognitive aspects, the trend of knowledge on Artificial Intelligence (AI) in this digital era we are living in has affected the readiness students need in facing their future (Hasibuan et al., 2022). AI knowledge and skills refer to the capacity of a person to strategically generate value by integrating AI technologies into organizational process capabilities and knowledge management (Firmansyah et al., 2025). We have seen how generative AI tools are spreading in education through teacher generation and integration by the teachers themselves, which is also a direct influence on student learning performance and creativity (Şimşek et al., 2025). Those people with a high level of AI awareness could utilize these tools as the levers to adapt learning, process hard data and automate tasks at routine jobs, and focus on more strategic aspects (Porkodi et al., 2025). In addition, a favorable view of AI leads to lower technology-based change anxiety, making them more learning agile (Gerçek & Özveren, 2025). AI Awareness can be regarded as a modern catalyst which proliferates to enhance students’ learning agility due to their enhanced cognitive flexibility, and the provision of new realms for exploration (Firmansyah et al., 2025).

Past research has examined learning agility, self-regulating ability and entrepreneurship separately, but the combination of these constructs along with technological knowledge in a high school setting has not been well-researched. Whereas most available research concentrates on samples of managers or university students (Firmansyah et al., 2025; Shavaran et al., 2025), the developmental pattern and ‘digital native’ status of high school pupils present a new dimension to this complex chain (Kaya, 2023). Students with good self-regulation skills generally utilize AI-enabled technologies more consciously and efficiently; this allows them to adapt the latest technology to their learning processes accordingly (Şimşek et al., 2025). This condition also suggests a causal sequence in which self-regulation develops AI awareness and AI awareness improves learning agility on a spectrum from entrepreneurial characteristics to learning agility (Prihandono et al., 2025). Cultivating these meta-competencies in high school students will equip them not only to be successful academically but also for the innovation-centric future world of business (Ab Jalil et al., 2022).

Despite these advances, previous studies have predominantly focused on isolated relationships among entrepreneurship, self-regulation, AI-related competencies, and learning agility, limiting a comprehensive understanding of how these variables operate together. Furthermore, evidence regarding the mediating mechanisms linking entrepreneurial tendencies to learning agility remains scarce, particularly among high school students. To address these limitations, the present study proposes a serial mediation model that integrates self-regulation and AI awareness within a single explanatory framework. By examining both cognitive and technological pathways simultaneously, the study provides a more comprehensive explanation of how entrepreneurial tendencies contribute to learning agility.

### 1.1. The Relationship Between Individual Entrepreneurship Tendencies and Learning Agility

Entrepreneurship of the individual is described as how individuals identify new opportunities, take risks and attempt to create novelty through innovative ideas, while learning agility involves a rapid behavioral change based on experiences (Haring et al., 2016). The link between these two concepts is the cognitive process by which an individual converts his/her psychological resources into tangible innovation results. Research in 2025 demonstrated that learning agility is a moderator, which moderates the direct relationship between how individuals perceive their status within an organization (e.g., feeling belonged and valued) and their internal entrepreneurial behaviors (Tayyab & Sharif, 2025). Consistent with these results, employees’ self-reported learning agility is higher when they feel a stronger attachment to the employer because it enables them to initiate action, experiment with new things and recover from setbacks in times of uncertainty. In this respect, learning agility serves as a major catalyst that can aid individuals in translating psychological belonging into constructive and active entrepreneurship behavior.

There is an embedded readiness to change in the dynamic of the entrepreneurial process, which further emphasizes the importance of learning agility. This ability allows people to shed previous and obsolete information (unlearning) and learn new skills quickly for dealing with novel environments or situations (Haring et al., 2016). A “adaptive orientation” is how Milani et al. (2024) define learning agility, focusing on one’s ability to bounce back and reorient in the context of complex situations and adversities. In this sense, the entrepreneurs become less sensitive to market dynamics and may achieve a competitive edge (Liu et al., 2002). Especially with the disorder caused by technological changes and global events, entrepreneurs have found it necessary not only to support existing businesses but also to find additional means of doing so. In this line, learning agility is the cornerstone of the ambidextrous innovation that balances both exploitation (doing things right) and exploration (doing the right thing) activities (Wang & Chugh, 2014; Tayyab & Sharif, 2025).

Entrepreneurship is fundamentally viewed as learning, and experiential learning predominates (Cope, 2005). According to Huovinen and Tihula (2008), entrepreneurs learn from their failures and nontrivial learning events not only from success. Likewise, people with high learning agility are more likely to be critical of, or second-guess, their experiences and learn from previous failures, resulting in heightened future entrepreneurial success (Kaya, 2023). In fact, Huovinen and Tihula (2008) is able to conclude that portfolio entrepreneurs transfer learning from earlier experiences with failure towards their new ventures and by doing so keep adding to their entrepreneurial knowledge. Furthermore, learning agility is highly associated with an individual’s “mastery orientation”. It has been linked to the interest in learning new abilities and further personal competences of an individual (Milani et al., 2024). In contrast, mental flexibility entails the capacity to come up with new ideas while thinking outside of the box in ambiguous conditions (Haring et al., 2016). This kind of mental agility is also required in entrepreneurship, where you need to recognize and evaluate opportunities. For instance, entrepreneurs who best reflect on an intuitive learning style are better at abstract thinking and expansion of opportunities, whereas those who prefer a sensing learning style process more concrete information. The effective entrepreneurial activity should draw on the flexible balancing of both learning styles (Bagheri & Pihie, 2010; Wang & Chugh, 2014).

On the other hand, individual entrepreneur-ship does not exist merely in terms of behaviors on which an individual acts alone, but also relates to norms for interacting with others who share similar goals and values, for working collaboratively, and even learning socially. “people agility” in this case, is same as the individual flexibility to be open to people from different cultures and backgrounds and able to learn from them (Kaya, 2023). Studies demonstrate that human agility is one of the most influential dimensions for continued performance enhancement (Haring et al., 2016). In addition, agile teaching formats in entrepreneurship education contribute to boosting students’ entrepreneurial skills such as teamwork, communication, and social competencies (Fischer et al., 2022).

Finally, it can be concluded that the relationship between personal entrepreneurship and learning agility is multi-faceted and strong. This paper identifies learning agility as a core competency that allows entrepreneurs to deal with uncertainty, learn from failure and turn creative ideas into actionable strategies. In fact, Tayyab and Sharif’s (2025) results underline that the sense of belonging of employees to the organization is converted into internal entrepreneurial behavior by learning agility, which itself is one of the key drivers for the success of entrepreneurship.

### 1.2. The Role of Self-Regulation

Self-regulation is the process by which an individual systematically organizes, monitors, and adapts their thoughts, feelings, and actions to cyclically changing conditions to achieve personal goals (Lawson et al., 2019; Rasyid, 2023; Winters et al., 2008). This capacity is a proactive activity that transforms the individual from a passive object of learning experiences into the manager and responsible party of their own development process (Elhusseini et al., 2022; Rasyid, 2023; Weinstein et al., 2011). Self-regulation consists of planning, performance monitoring, and reflective evaluation stages, enabling the individual to transform their internal motivation into concrete actions (Sáez-Delgado et al., 2022; Weinstein et al., 2011).

Self-regulation plays a critical mediating role in the relationship between proactive personality, a reflection of individual entrepreneurship, and learning agility, the outcome variable. Proactive people create opportunities, they challenge and move beyond the constraints in their environment, focusing on the future; however, this potential can only be transferred into learning agility by how well people manage their cognitive processes and emotions (Shavaran et al., 2025; Li & Chalermvongsavej, 2025). Self-regulation at this time serves as a strategic “engine” and “glue” transmuting the individual’s entrepreneurial energy into flexible adaptational capacities (Weinstein et al., 2011; Shavaran et al., 2025). Studies have shown that self-regulation capacity might explain 43.1% of variance in cognitive agility and that it directly enhances agile maneuverability under uncertain circumstances (Jøsok et al., 2019).

This instrumental role of self-regulation is manifested in emotion regulation and strategic control processes. Entrepreneurial people can overcome these barriers with their use of adaptive self-regulation strategies, such as “cognitive reappraisal”, and preserve their cognitive ability and enhance learning agility (Shavaran et al., 2025). This process promotes mental health and flexibility by contributing to the ability of a person to “bounce back” (buoyancy) during academic stress and everyday disruptions (Huang & Kou, 2025; Ragusa et al., 2023). In addition, self-regulation redirects the individual’s preactivity potential from maladaptive activities such as procrastination and channels this into creative problem-solving and strategic planning cycles (Hidajat, 2023; Ragusa et al., 2023). In the end, self-regulation serves as the overarching mechanism to transfer opportunity-recognition and challenge-orientation of proactive individuals into “learning agility” (Shavaran et al., 2025; Kim et al., 2023).

### 1.3. The Role of Artificial Intelligence Awareness

In today’s Volatile, Uncertain and Complicated (VUCA) world, high school students must learn how to release their own entrepreneurial potential, develop preparation for the future and their careers (Ab Jalil et al., 2022; Gerçek & Özveren, 2025). In this procedure, AI awareness as the mediating strategy and cognitive driver between students’ entrepreneurial mindset and learning agility was evident (Porkodi et al., 2025; Prihandono et al., 2025). Entrepreneurial vision students and those with a creative self-efficacy view AI technologies as not only a technical tool but also an innovative tool, which will assist them in the actualization of their projects and competitive advantage (Gerçek & Özveren, 2025; Alshammari & Alshammari, 2026). This knowledge and technological literacy accelerate the speed of students in recognizing, combining, and applying new knowledge to create resilience and flexibility vis-à-vis technological change, which eventually enhances student learning agility (Mardiana, 2025; Firmansyah et al., 2025).

When learning agility becomes the dependent variable in this model, it is evident that such an agility (empowered by AI awareness) enables students to engage with proactive career behaviors and ultimately become much better prepared to face the business world in the Industry 4.0 setting (Ab Jalil et al., 2022; Gerçek & Özveren, 2025). High school students with higher levels of learning agility have made use of the AI tools more easily and effectively, which in turn has expedited technological integration into entrepreneurial activities (Şimşek et al., 2025). AI talents work like a tool to turn the knowledge of one into a human force of agility, so that entrepreneurial students can transform their technological experience into personal competitive strength (Firmansyah et al., 2025; Prihandono et al., 2025). As such, while entrepreneurial competences at high school become the “prime mover”, AI awareness is potentially a limiting factor that translates the energy into process performance under novel and challenging conditions, i.e., learning agility (Alshammari & Alshammari, 2026; Porkodi et al., 2025).

### 1.4. Current Study

The current study focuses on learning agility, a critical meta-competence for high school students’ academic success and future readiness in today’s volatile, uncertain, complex, and ambiguous world (Ab Jalil et al., 2022; Shavaran et al., 2025). In the literature, learning agility is defined as the ability to learn quickly from experiences and apply this knowledge flexibly to new, challenging conditions, and is seen as a fundamental “survival tool” for individuals’ professional sustainability (Milani et al., 2024; Lawson et al., 2019). To this extent, the research aims to evaluate whether individual entrepreneurial tendency characterized by its innovative, proactive and risk-taking tendencies, evokes students’ speed of organizing and adapting the new information. Entrepreneurship at the individual level is not limited to establishing a new business, but it is also considered a “catalyst” which activates cognitive resources and helps in addressing the complex problems and subsequently enhances learning agility. For high school students, entrepreneurial tendency may be reflected in behaviors such as generating original ideas, taking initiative in academic tasks, seeking alternative solutions to learning challenges, and proactively adapting to new educational demands.

The novelty of the research is that it examines the mediating effects of two cognitive and technological factors, self-regulation and AI awareness, in the connection between entrepreneurial propensity and learning agility in an all-inclusive model. The third task-related factor in this model, self-regulation or students’ capability to organize, control and evaluate their own learning, appears to be a strategic cognitive control skill (Winters et al., 2008; Weinstein et al., 2011). Studies have revealed that learning agility can be more pronounced for those high in self-regulation because they display cognitive flexibility better (Jøsok et al., 2019; Huang & Kou, 2025). A further moderating construct is artificial intelligence knowledge, which may be perceived as an “innovation lever” to translate technological exposure into a tangible competitive advantage (Prihandono et al., 2025; Alshammari & Alshammari, 2026). Accordingly, this research seeks to clarify how entrepreneurial tendency contributes to students’ success in novel and unpredictable situations through self-regulation and AI awareness as sequential mediating variables. Students with stronger self-regulation skills are more likely to use and evaluate AI-related technologies effectively, thereby developing greater AI awareness, which may subsequently enhance learning agility.

The present study makes several contributions to the literature. First, it integrates entrepreneurial tendency, self-regulation, artificial intelligence awareness, and learning agility within a single explanatory framework. Second, it extends previous research by examining the mediating role of AI awareness, a construct that has received limited attention in learning agility research. Third, unlike many previous studies conducted with university students or working adults, this study focuses on high school students, an understudied population despite their increasing exposure to digital technologies. Finally, by testing a serial mediation model, the study provides a more comprehensive understanding of the cognitive and technological mechanisms through which entrepreneurial tendency contributes to learning agility. Accordingly, our hypotheses are:

**H_1_:**  *Individual entrepreneurship positively and significantly predicts learning agility.*

**H_2_:**  *Self-regulation and artificial intelligence awareness sequentially mediate the relationship between individual entrepreneurship and learning agility.*

**H_3_:**  *Self-regulation positively predicts artificial intelligence awareness.*

## 2. Method

### 2.1. Participants

A total of 564 high school students participated in the study. Of these, 55.0% (*n* = 310) were female, and 45.0% (*n* = 254) were male. By grade level, 16.0% (*n* = 90) were in Year 10, 14.7% (*n* = 83) were in Year 11, and 69.3% (*n* = 391) were in Year 12. Participants’ ages ranged from 14 to 19 years (*M* = 17.02, *SD* = 1.28). The age distribution was as follows: 7.3% (*n* = 41) were 14 years old, 7.3% (*n* = 41) were 15, 12.2% (*n* = 69) were 16, 25.7% (*n* = 145) were 17, 44.5% (*n* = 251) were 18, and 3.0% (*n* = 17) were 19 years old. The majority of participants were 18 years old.

Assuming a medium effect size (f^2^ = 0.15) as recommended by Cohen (1988), an α = 0.05 significance level, 0.80 statistical power, and three predictor variables, the minimum required sample size was calculated to be 77 participants. As 564 students participated in the study, the sample size was more than sufficient and provided strong statistical power for the analyses (Chen & Liu, 2019). In addition, a sensitivity analysis conducted using G*Power 3.1 indicated that, with a sample size of 564, α = 0.05, power = 0.95, and four predictors, the study was capable of detecting a minimum effect size of f^2^ = 0.033, indicating sufficient power to detect even small effects.

### 2.2. Measures

#### 2.2.1. Individual Entrepreneurship Tendencies Scale

The scale measuring high school students’ individual entrepreneurial tendencies, developed by Kurniawan et al. (2019, 2021), was adapted into Turkish by Demiralp and Turunç (2025). As part of the construct validity assessment, exploratory factor analysis revealed that the scale has a three-dimensional structure (innovativeness, risk-taking, and proactiveness-competitiveness) explaining 63.50% of the total variance. Confirmatory factor analysis also confirmed that this model is statistically valid. The Cronbach’s alpha value, which is the general reliability coefficient of the scale, was calculated as 0.93, while the values of the sub-dimensions ranged between 0.89 and 0.93 (Demiralp & Turunç, 2025). The scale, consisting of a total of 24 items, is a 5-point Likert scale rated from 1 to 5 (Demiralp & Turunç, 2025).

#### 2.2.2. Learning Agility Scale for High School Students

The Learning Agility Scale for High School Students, developed by Çetin (2023) as part of a doctoral dissertation, was used to assess students’ learning agility. Exploratory factor analysis conducted during the scale development process revealed a four-factor structure explaining 54.105% of the total variance, and this structure was supported by confirmatory factor analysis results (RMSEA = 0.08, CFI = 0.90) (Çetin, 2023). In reliability analyses, the Cronbach’s alpha coefficient for the overall scale was 0.79, while the reliability coefficients of the sub-dimensions ranged from 0.59 to 0.79. Although one sub-dimension yielded a relatively lower reliability coefficient (α = 0.59), this may be attributed to the limited number of items within that factor. As noted by Cortina (1993), lower reliability coefficients may be expected in factors containing relatively few items. The scale consists of 18 items rated on a five-point Likert scale.

#### 2.2.3. Self-Regulation Scale for Adolescents

The Self-Regulation Scale for Adolescents was developed by Kaşıkcı and Öğülmüş (2023) to measure the general skills of high school students. The exploratory factor analysis conducted for construct validity revealed that the scale is unidimensional and explains 51% of the variance. Confirmatory factor analysis also determined that the model fit (CFI = 0.93, RMSEA = 0.089) is at a good level (Kaşıkcı & Öğülmüş, 2023). The scale’s internal consistency coefficient, Cronbach’s Alpha, was calculated as 0.90, while McDonald’s Omega and composite reliability (CR) values were calculated as 0.89. The scale consists of 11 items and uses a 5-point Likert-type rating scale (Kaşıkcı & Öğülmüş, 2023).

#### 2.2.4. Artificial Intelligence Awareness Scale for Adolescents

The Artificial Intelligence Awareness Scale for Adolescents (AIASA), developed by Öner et al. (2025), aims to measure young people’s level of awareness regarding this technology (Öner et al., 2025). According to exploratory factor analysis data, the scale presents a four-dimensional structure (interest, perceived benefit, usage knowledge, and perceived risk) explaining 60.42% of the total variance, and this structure shows a high level of fit with confirmatory factor analysis indices (CFI = 0.99, GFI = 0.99). In reliability studies, the Cronbach Alpha coefficient for the entire scale was found to be 0.931, while the reliability of the sub-dimensions ranged from 0.786 to 0.909. The scale, prepared in a six-point Likert type, consists of 20 items in total (Öner et al., 2025).

### 2.3. Procedures

The sample consisted of a group of high school students invited to participate in the study at two state high schools in eastern Turkey. Before data collection, ethical approval was obtained from the Scientific Research Ethics Committee of Ağrı İbrahim Çeçen University (Decision No: 482, 27 November 2025), and written permission was obtained from the school administration. Participation in the study was voluntary. Students, school staff, and parents were informed about the study, and data were collected with informed consent.

Data were collected via paper-and-pencil administration in a closed classroom setting, where participants completed a demographic form along with the relevant measurement scales. Participation was entirely voluntary. School children and parents were informed about the purpose of the study, and written consent was obtained. Participants were informed that their responses would be anonymous and used solely for research purposes. The questionnaires were administered to each volunteer within approximately 15–20 min. The procedures followed were in accordance with the ethical standards of the Helsinki Declaration.

### 2.4. Data Analysis

Prior to the main analyses, regression assumptions were examined. Normality was assessed using skewness and kurtosis values, and multicollinearity was evaluated using variance inflation factor (VIF) values. All VIF values were below the recommended thresholds, indicating no serious multicollinearity concerns. No substantial violations of regression assumptions were detected (Lin, 2008). Indirect effects were tested using the bootstrapping procedure with 5000 resamples and a 95% confidence interval. Pearson correlation coefficient analysis was performed to calculate the relationships between individual entrepreneurial inclination, self-regulation, artificial intelligence awareness, and agility. In the second stage, regression analysis revealed that individual entrepreneurial inclination, self-regulation, and artificial intelligence awareness could predict learning agility. In the third stage, the study’s mediation processes showed that individual entrepreneurial inclination indirectly mediated learning agility through self-regulation and artificial intelligence awareness. In the fourth stage, bootstrap confidence intervals were used to assess the significance of mediation in order to determine whether mediation was meaningful (Hayes, 2013). The PROCESS Macro applied the mediation model of this study in Model 6, and the 95% confidence interval of the indirect effect was used to determine its significance (Preacher & Hayes, 2008).

## 3. Results

### 3.1. Preliminary Analyses

Table 1 shows the descriptive statistics, skewness-kurtosis values, and correlation coefficients of the variables. All variables in this study showed a normal distribution. Skewness values ranged between ±1.5, while kurtosis values also remained within ±1.5, indicating acceptable normality (Tabachnick et al., 2013). Cohen’s (1988) classification was used to interpret the strength of the correlation coefficients (r = 0.10, low; r = 0.30, moderate; r = 0.50, strong).

According to the results, Individual Entrepreneurship Propensity showed low but significant positive correlations with Self-Regulation (r = 0.168, *p* < 0.001), Artificial Intelligence Awareness (r = 0.183, *p* < 0.001), and Learning Agility (r = 0.274, *p* < 0.001). Self-Regulation showed a moderate and positive correlation with Artificial Intelligence Awareness (r = 0.418, *p* < 0.001) and a low to moderate correlation with Learning Agility (r = 0.390, *p* < 0.001). Finally, a strong, positive, and significant relationship was found between Artificial Intelligence Awareness and Learning Agility (r = 0.599, *p* < 0.001). These findings indicate that all variables are positively related with varying degrees of relationship strength.

### 3.2. Serial Mediation Analysis

A serial multiple mediation analysis was conducted to examine the mediating roles of Self-Regulation (M1) and Artificial Intelligence Awareness (M2) in the relationship between Individual Entrepreneurship Propensity (X) and Learning Agility (Y). In this model, Individual Entrepreneurship Propensity was defined as the independent variable, Learning Agility as the dependent variable, while Self-Regulation and Artificial Intelligence Awareness served as first- and second-order mediators, respectively.

**Figure 1:**
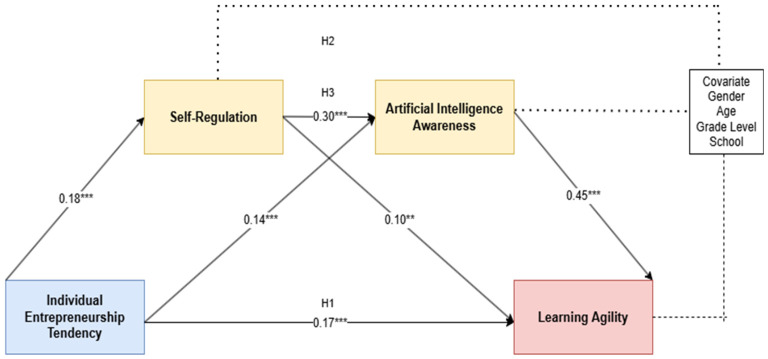
Serial mediation model with standardized coefficients (β). Values represent standardized coefficients. Colors indicate variable types (independent, mediating, and dependent variables), and dotted lines indicate control variables. ** *p* < 0.01, *** *p* < 0.001.

As shown in Figure 1, all path coefficients were statistically significant. The results revealed a significant serial mediation effect, indicating that individual entrepreneurial inclination predicted self-regulation, self-regulation predicted artificial intelligence awareness, and ultimately led to learning agility. Control variables such as gender, age, class level, and school were included in the model to account for their potential effects.

According to the sequential mediation analysis results, individual entrepreneurial inclination significantly and positively predicted self-regulation (β = 0.18, *p* < 0.001) and artificial intelligence awareness (β = 0.14, *p* < 0.001). Furthermore, individual entrepreneurship also has a direct and significant positive effect on learning agility (β = 0.18, *p* < 0.001).

The analysis revealed that self-regulation significantly and positively predicted artificial intelligence awareness (β = 0.30, *p* < 0.001) and had a smaller but significant positive effect on learning agility (β = 0.10, *p* = 0.005). Additionally, artificial intelligence awareness strongly and positively predicted learning agility (β = 0.46, *p* < 0.001).

These findings indicate that individual entrepreneurial inclination influences learning agility both directly and indirectly through two mediators: self-regulation and artificial intelligence awareness. Specifically, a strong indirect path through both mediators shows a significant serial mediation effect; in this effect, higher entrepreneurial inclination leads to better self-regulation and artificial intelligence awareness, which in turn increases learning agility. Control variables such as gender, age, class level, and school were included in the model, but their effects were not significant, except for school (β = 0.16, *p* < 0.001), which was positively related to learning agility.

Following preliminary analyses, a sequential mediation analysis (PROCESS Model 6) was conducted to examine whether self-regulation and AI awareness mediated the relationship between individual entrepreneurial inclination and learning agility (Table 2). The analysis revealed that individual entrepreneurship significantly and positively predicted self-regulation (B = 0.090, *p* < 0.001) and artificial intelligence awareness (B = 0.116, *p* < 0.001). Furthermore, it had a significant direct effect on learning agility (B = 0.154, *p* < 0.001). Moreover, self-regulation significantly predicted artificial intelligence awareness (B = 0.498, *p* < 0.001) and had a smaller but significant effect on learning agility (B = 0.177, *p* = 0.005). AI awareness strongly and positively predicted learning agility (B = 0.481, *p* < 0.001).

The total effect of individual entrepreneurship on learning agility (B = 0.247, *p* < 0.001, 95% CI [0.184, 0.311]) and the total indirect effect (B = 0.093, 95% CI [0.056, 0.132]) were significant. Among specific indirect paths, the indirect effect via self-regulation (B = 0.016, 95% CI [0.004, 0.032]), the indirect effect via AI awareness (B = 0.056, 95% CI [0.026, 0.085]), and the sequential path from self-regulation to AI awareness (B = 0.022, 95% CI [0.011, 0.035]) were statistically significant.

These findings indicate that individual entrepreneurial inclination affects learning agility both directly and indirectly through improvements in self-regulation and artificial intelligence awareness. The overall model explained 16.8% of the variance in self-regulation (R^2^ = 0.168), 25.2% of the variance in AI awareness (R^2^ = 0.252), and 43.2% of the variance in learning agility (R^2^ = 0.432). According to Cohen’s (1988) criteria, the explained variance in learning agility corresponds to a large effect size (f^2^ = 0.76). Among the covariates, school (high school) significantly predicted learning agility (B = 4.43, *p* < 0.001), while gender, age, and grade level were not significant predictors.

The results showed several significant indirect effects, as the 95% bias-corrected confidence intervals excluded zero (Table 3). The strongest pathway was through artificial intelligence awareness. Indirect effects via self-regulation, as well as the sequential path through self-regulation followed by artificial intelligence awareness, were also statistically significant. Standardized indirect effects and confidence intervals for each path are presented in Table 3.

## 4. Discussion

The findings indicate that individual entrepreneurial tendencies in high school students are positively associated with learning agility. This finding suggests that students with an entrepreneurial mindset tend to report greater capacity to cope with uncertain situations and learn quickly from their experiences. In the literature, entrepreneurship is defined not merely as a process of starting a business, but as an experimental and dynamic process of “learning to learn” that views mistakes as learning opportunities. Liu et al. (2002) argue that entrepreneurial culture promotes learning orientation and forms the basis of organizational/individual success. Similarly, a strong correlation has been found between proactive personality traits (as a dimension of entrepreneurship) and learning agility, with proactive individuals having a higher capacity to identify new opportunities. Fischer et al. (2022) state that entrepreneurship education develops students’ social skills, such as reflection and resilience, making them more “agile” learners. In this context, it can be said that high school students with high entrepreneurial tendencies behave more proactively in their learning processes and adapt more quickly to new and challenging conditions.

The finding in line with the second hypothesis of the study is that self-regulation and artificial intelligence awareness play a serial mediation role in the relationship between entrepreneurial inclination and learning agility. This model suggests that entrepreneurial tendency is positively associated with self-regulation and AI awareness, and that these variables are associated with learning agility within the proposed sequential framework. Weinstein et al. (2011) argue that SR functions both as a “motor” and “glue” of SRL, allowing students to regulate their cognitive resources. Research by Firmansyah et al. (2025) highlights that competence in artificial intelligence serves as a crucial link between knowledge management and learning agility. Furthermore, Şimşek et al. (2025), metacognitive self-regulation skills are an important determinant for the adoption of artificial intelligence technologies and related performance quality. Hasibuan et al. (2022) revealed that those students who possessed strong self-regulation abilities in a digital learning environment showed increased metacognition, which reflected positively on their learning performance. These findings suggest that technological literacy and problem-solving skills may be associated with higher levels of learning agility, so that all people can get ready to face various challenges in a millennial digital age movement (Mardiana, 2025).

The results showed that there was a positive and significant relation between self-regulation variables and covariance with awareness of artificial intelligence. Students capable of planning, monitoring, and regulating their learning when leading in complex technologies like AI are more likely to understand the technology adequately, as well as adopt it. Şimşek et al. (2025) suggest that for individuals high in self-regulation capability, adopting artificial intelligence-embedded tools is associated with lower cognitive load and greater perceived usefulness. Hidajat (2023) mentions that self-regulation skills are associated with creative thinking or new knowledge discovery process of students, as well as interaction with technological tools. Zheng et al. (2023) claim that self-regulation in online learning environments further involves a direct enhancement of the student’s engagement with and perception of technology. Winters et al.’s (2008) study on the relationship between technology use and academic achievement in computer-based learning environments is self-management, as was emphasized by DiCerbo et al. Finally, Jøsok et al. (2019) confirm such a link, where self-regulation skills are positively associated with cognitive agility needed for navigating within complex and hybrid discipline environments. Although the correlation between AI awareness and learning agility was relatively strong, multicollinearity diagnostics indicated no statistical concern. This relationship may reflect the conceptual proximity between students’ awareness of emerging technologies and their capacity to adapt to new learning demands, while still representing distinct constructs.

All findings obtained specifically for high school students indicate that, in a future dominated by academic and professional uncertainties, individual entrepreneurial tendencies alone are not sufficient; this potential must be supported by meta-competencies such as self-regulation and artificial intelligence awareness (Li & Chalermvongsavej, 2025). The findings suggest that high school students with an entrepreneurial mindset primarily reflect their proactive approaches in their ability to plan and monitor their own learning processes (self-regulation), and that this internal discipline leads them to view artificial intelligence technologies, which are a necessity of the age, as strategic learning tools (AI awareness) (Şimşek et al., 2025). Students with developed self-regulation skills experience less cognitive load when using artificial intelligence tools and leverage these technologies to enhance their learning agility, moving them away from traditional learning models and making them “future-read” (Ab Jalil et al., 2022; Gerçek & Özveren, 2025). Consequently, the observed associations among entrepreneurship, self-regulation, AI awareness, and learning agility suggest that these factors may be linked to students’ capacity to learn from their experiences and navigate the complex demands of an increasingly digitalized world (Mardiana, 2025; Porkodi et al., 2025). The persistence of a significant direct effect of entrepreneurial tendency on learning agility, alongside significant indirect effects, suggests that self-regulation and AI awareness partially mediate this relationship rather than fully explaining it.

### 4.1. Implications

This article contributes to the literature on learning agility, especially with attention being paid to ‘digital native’ (Kivunja, 2014) in transitional developmental stages such as high schools. Most existing research has focused on samples of managers or university students (Milani et al., 2024; Li & Chalermvongsavej, 2025). This work informs that meta-competencies are formed at a very early age. The transfer of the Knowledge-Based View and Dynamic Competence Theory to the education system has shown that technology awareness (AI awareness) is no longer a tool but rather a strategic bridge of transposition from cognition (self-regulation) towards agility (Alshammari & Alshammari, 2026; Firmansyah et al., 2025). Moreover, the result that entrepreneurial inclination impacts learning speed in an indirect way by sequential cognitive and technological means instead of a direct effect indicates the necessity to establish an all-inclusive model in educational psychology (Mardiana, 2025; Şimşek et al., 2025).

### 4.2. Limitations

There are certainly some limitations to this study despite its valuable findings. First, given the cross-sectional nature of the study, it is not possible to establish causal relationships among the variables; therefore, longitudinal designs are needed to examine how learning agility develops over time. Second, the use of self-report measures may have introduced response biases, such as social desirability bias. Third, because the sample consisted of students from selected Turkish high schools, the findings may not be generalizable to other cultural or socio-economic contexts. In addition, potentially influential contextual factors, such as family support, school technological infrastructure, and students’ academic self-efficacy, were not included in the model. Future studies could enrich these findings by incorporating qualitative data (e.g., interviews and observations) and examining the direct implications of emerging technologies, including generative artificial intelligence, for learning processes. Future research may also test the proposed model across different educational levels and cultural contexts to improve generalizability. Although the present study relied on traditional frequentist statistical analyses, future studies may benefit from employing Bayesian analytical approaches, which can provide additional evidence regarding parameter estimation and model uncertainty. Finally, longitudinal and experimental designs may offer stronger evidence regarding the relationships among entrepreneurial tendency, self-regulation, AI awareness, and learning agility.

### 4.3. Conclusions

This study contributes to understanding the personal and cognitive factors associated with learning agility among high school students. The findings indicate that entrepreneurial tendency is positively associated with learning agility, suggesting that students with stronger entrepreneurial tendencies tend to report higher levels of adaptability and a greater capacity to cope with uncertainty. Importantly, these associations were observed both directly and indirectly through self-regulation and AI awareness within the proposed sequential model. Entrepreneurial tendency was positively associated with self-regulated learning (e.g., planning and monitoring), and self-regulation was positively associated with AI awareness. Furthermore, self-regulation and AI awareness were both positively associated with learning agility. The association between self-regulation and AI awareness may indicate that students with stronger self-regulatory skills tend to report greater awareness of AI technologies in educational contexts. The proposed model explained 43.2% of the variance in learning agility, 25.2% in AI awareness, and 16.8% in self-regulation. Overall, the findings suggest the potential value of educational programs that integrate entrepreneurship, self-management, and digital competencies in supporting students’ adaptation to increasingly complex and innovation-oriented learning environments.

## Figures and Tables

**Table 1:** Descriptive statistics and correlations between variables.

Variables	1	2	3	4
1. Individual Entrepreneurship Tendencies	1	0.17 ***	0.18 ***	0.27 ***
2. Self-Regulation		1	0.42 **	0.39 ***
3. Artificial Intelligence Awareness			1	0.60 ***
4. Learning Agility				1
Average	87.14	37.89	66.43	59.08
Standard deviations	15.68	7.85	12.89	13.49
Skewness	−0.60	−0.21	−0.02	−0.02
Kurtosis	−0.06	−0.93	−0.84	−1.05

Note. ** = *p* < 0.01; *** = *p* < 0.001.

**Table 2:** Unstandardized coefficients for the mediation model.

	M_1_ (Self-Regulation)				M_2_ (Artificial Intelligence Awareness)				Y (Learning Agility)			
Predictor	B	SE	t	*p*	B	SE	t	*p*	B	SE	t	*p*
X (Individual Entrepreneurship Tendencies)	0.09	0.02	4.6	<0.001	0.11	0.03	3.75	<0.001	0.15	0.02	5.39	<0.001
M_1_ (Self-Regulation)	—	—	—	—	—	—	—	—	0.17	0.06	2.81	0.05
M_2_ (Artificial Intelligence Awareness)	—	—	—	—	—	—	—	—	0.48	0.03	12.43	<0.001
Constant	22.82	5	4.56	<0.001	18.92	7.94	2.38	0.018	−3.25	7.28	−0.45	0.656
	R^2^ = 0.16 F(5, 558) = 22.48 *p* < 0.001				R^2^ = 0.25 F(6, 557) = 31.21 *p* < 0.001				R^2^ = 0.43 F(7, 556) = 60.39 *p* < 0.001			

Note. — = Not applicable.

**Table 3:** Standardized indirect effects and 95% bias-corrected confidence intervals.

Indirect Path	Standardized Effect	BootSE	95%	
			Bootstrap LLCI	Boot ULCI
Individual Entrepreneurship Tendency → Self-Regulation → Learning Agility	0.02	0.01	0.00	0.04
Individual Entrepreneurship Propensity → Artificial Intelligence Awareness → Learning Agility	0.06	0.02	0.03	0.10
Individual Entrepreneurship Propensity → Self-Regulation → Artificial Intelligence Awareness → Learning Agility	0.03	0.01	0.01	0.04

## Data Availability

The data that support the findings of this study are available from the MY upon reasonable request.
